# Somatic retrotransposition is infrequent in glioblastomas

**DOI:** 10.1186/s13100-016-0077-5

**Published:** 2016-11-11

**Authors:** Pragathi Achanta, Jared P. Steranka, Zuojian Tang, Nemanja Rodić, Reema Sharma, Wan Rou Yang, Sisi Ma, Mark Grivainis, Cheng Ran Lisa Huang, Anna M. Schneider, Gary L. Gallia, Gregory J. Riggins, Alfredo Quinones-Hinojosa, David Fenyö, Jef D. Boeke, Kathleen H. Burns

**Affiliations:** 1 Molecular Biology and Genetics, Johns Hopkins University School of Medicine, Baltimore, MD USA; 2 Department of Pathology, Johns Hopkins University School of Medicine, Miller Research Building (MRB) Room 447, 733 North Broadway, Baltimore, MD 21205 USA; 3 McKusick-Nathans Institute of Genetic Medicine, Johns Hopkins University School of Medicine, Miller Research Building (MRB) Room 447, 733 North Broadway, Baltimore, MD 21205 USA; 4 Center for Health Informatics and Bioinformatics, New York University Langone Medical Center, New York, NY USA; 5 Institute for Systems Genetics, New York University Langone Medical Center, ACLSW Room 503, 430 East 29th Street, New York, NY 10016 USA; 6 Department of Neurosurgery, Johns Hopkins University School of Medicine, Baltimore, MD USA; 7 Present address: Yale University, New Haven, CT USA; 8 Present address: L.E.K. Consulting, Boston, MA USA; 9 Present address: BioNTech AG, Mainz, Germany; 10 Present address: Mayo Clinic, Jacksonville, FL USA

**Keywords:** LINE-1, Retrotransposition, Cancer, Glioblastoma

## Abstract

**Background:**

Gliomas are the most common primary brain tumors in adults. We sought to understand the roles of endogenous transposable elements in these malignancies by identifying evidence of somatic retrotransposition in glioblastomas (GBM). We performed transposon insertion profiling of the active subfamily of Long INterspersed Element-1 (LINE-1) elements by deep sequencing (TIPseq) on genomic DNA of low passage oncosphere cell lines derived from 7 primary GBM biopsies, 3 secondary GBM tissue samples, and matched normal intravenous blood samples from the same individuals.

**Results:**

We found and PCR validated one somatically acquired tumor-specific insertion in a case of secondary GBM. No LINE-1 insertions present in primary GBM oncosphere cultures were missing from corresponding blood samples. However, several copies of the element (11) were found in genomic DNA from blood and not in the oncosphere cultures. SNP 6.0 microarray analysis revealed deletions or loss of heterozygosity in the tumor genomes over the intervals corresponding to these LINE-1 insertions.

**Conclusions:**

These findings indicate that LINE-1 retrotransposon can act as an infrequent insertional mutagen in secondary GBM, but that retrotransposition is uncommon in these central nervous system tumors as compared to other neoplasias.

**Electronic supplementary material:**

The online version of this article (doi:10.1186/s13100-016-0077-5) contains supplementary material, which is available to authorized users.

## Background

Glioblastomas (GBMs) are the most common malignant form of primary brain tumor in adults, and are typically fatal. These are histologically aggressive gliomas, categorized by the World Health Organization (WHO) as grade IV astrocytomas; they are hypercellular with frequent mitotic figures, vascular proliferation and pseudopalisading necrosis. Although morphologically indistinguishable, distinct primary and secondary types of GBM are recognized clinically. Primary GBMs arise *de novo*, and usually present as advanced cancers in patients over 50 years old. These are characterized genetically by amplification of epidermal growth factor receptor (*EGFR*), loss of heterozygosity (LOH) on chromosomes 10q and 17p, and phosphatase and tensin homologue (*PTEN*) mutation. Secondary GBMs arise from preexisting low-grade tumors over a period of a few years and are more common among younger patients. This class of tumors is characterized by mutations in isocitrate dehydrogenase 1 (*IDH1*) and p53 tumor suppressor genes as well *PDGFA* amplification [1, 2].

Activation of endogenous transposable elements as a mechanism of mutagenesis is being increasingly recognized in human tumors. Retrotransposons are a class of mobile genetic elements that use a ‘copy and paste’ mechanism to replicate in the genome through RNA intermediates. Among these, the autonomous Long INterspersed Element-1 (LINE-1 or L1s) are the most active elements in humans [3]. Recently, methods have been developed to identify LINE-1 sequences in human genomes that collectively underscore their ongoing potential for retrotransposition in the germline [4–9] and in malignancy [6, 10–18]. Recent studies have also implicated LINE-1 expression and activity in normal brain and in brain malformations and disease [19–22].

In this study, we mapped LINE-1 insertion sites in GBMs and matched blood samples using a targeted sequencing approach, Transposon Insertion Profiling (TIPseq). We profiled oncosphere cell lines derived from primary GBMs as compared to matched normal genomic DNA from the same patients [23]. We also used TIPseq to compare genomic DNA isolates from primary and secondary GBMs and from normal blood DNA from the same patients.

## Methods

### Consent statement

Blood and brain tumor tissue samples were obtained from glioma patients who underwent surgery at the Johns Hopkins Hospital under the approval of the Institutional Review Board (IRB) and with consent. This study included 7 primary GBM and 3 secondary GBM patients.

### Oncosphere cell cultures from primary glioblastoma tissue

Fresh primary glioblastoma tissue was dissociated enzymatically using TrypLE (Gibco). The homogenized tissue was passed through a narrow fire-polished Pasteur pipette and 40 μm cell strainer to obtain single cell suspension. Primary cells were then plated at a density of 1 × 10^5^ viable cells in 25-cm^2^ non-adherent flasks in DMEM/F12 medium supplemented with 20 ng/mL of human epidermal growth factor (EGF), and 10 ng/mL of human fibroblast growth factor (FGF). Oncospheres of approximately 100 μm were passaged and replated.

### Genomic DNA preparation and Vectorette PCRs

Genomic DNA from peripheral blood samples was isolated using the QIAamp DNA blood mini kit (Qiagen). Genomic DNA from tumor tissue and oncospheres was isolated by Trizol homogenization, phenol-chloroform-iso amyl alcohol extraction and ethanol precipitation. Aliquots of ~0.5–2 μg of genomic DNA from each sample were digested individually with six different restriction enzymes (*AseI, BspHI, BstYI, HindIII, NcoI, PstI*) generating fragments averaging 1–3 kb in length. Vectorette matched with restriction enzyme sticky-end sites were designed and ligated to the digested DNA fragments. Vectorette PCR was performed using *ExTaq* HS polymerase (Takara Bio) and a touch-down PCR program to generate amplicons spanning the transposon insertion end and the flanking unique genomic sequences [24, 25].

### Deep sequencing DNA libraries and quality control

Vectorette PCR products from each patient sample were pooled, purified and fragmented to an average length of 300 bp using a Covaris E210. TruSeq DNA Sample Preparation kit v2 (Illumina) was used for end-repair, A-tailing, index-specific adapter ligation and PCR enrichment. We size-selected our DNA fragments at ~450 bp using 2 % Size-Select E-gels (Life Technologies) prior to PCR. The enriched PCR products were purified and checked for quality control using an Agilent Bioanalyzer. The DNA libraries were pooled and submitted for single-end or paired-end deep sequencing with Illumina HiSeq 2000 platform either at Johns Hopkins high-throughput sequencing center or the HudsonAlpha Institute for Biotechnology (HudsonAlpha, Huntsville, AL). The sequencing batch, facility, indexes, barcodes and read lengths for each sample are provided in Additional file 1: Table S1.

### Computational analysis

Two analytical approaches were used. In the first, all trimmed reads (75–100 bp) were first aligned to the human reference genome (hg18) using Bowtie [26], and cisGenome was used for identifying peaks. The peaks were ranked based on the maximum base pair read coverage. Unmappable reads from the Bowtie alignment were used to identify the junctional reads. 35 bp from each of the 5′ and 3′ end of the unmappable reads were trimmed and aligned with the human reference genome. Reads aligning uniquely with only one end to the genome were extracted and grouped together according to the peak list using SAMtools. Those with at least six consecutive As or Ts were used for further analysis to enrich for transposon junctions. A maximum of 200 such junction reads per peak were used to generate the consensus sequences using multiple sequence alignment (MSA) and the bioperl AlignIO module. BLAT was used to compare each consensus sequence to both the hg18 reference genome and a 3′ LINE-1 sequence with polyA tail. Galaxy was used to identify lists of putative insertions occurring in *either* a blood or tumor sample for an individual and not both. The Integrative Genomics Viewer (IGV) was used to visualize the read alignments to the reference genome.

For secondary GBM TIPseq samples, a second machine learning algorithm analysis was also conducted using paired-end read samples. Low quality sequences, base pairs, and vectorette sequences were trimmed using Trimmomatic software [27]. Qualified read pairs were aligned to an L1Hs-masked reference genome (hg19) and the L1Hs consensus sequence using Bowtie2 software. Candidate insertion sites were identified as peaks with at least one junction-containing read pair. The machine learning model was trained on known LINE-1 insertions using five sequencing features, namely the peak width and depth; variant index for reads mapping in the peak interval; the polyA tail purity; and the number of junction reads. The trained model was used to predict probabilities of the candidate insertions being the true insertion sites. This pipeline, TIPseqHunter, will be reported in more detail elsewhere (Tang, Z., et al. in review).

### PCR validations and Sanger sequencing

Primers were designed to flank putative LINE-1 insertion sites using Primer3 software. PCR products with insertions were cut out of the gel and DNA was extracted using a QIAquick Gel extraction kit (Qiagen). The purified PCR products were then sent for Sanger sequencing to obtain the 5′ and 3′ junction coordinates, length and orientation of the inserted L1, and target site duplication.

### Copy number variation (CNV) and loss of heterozygosity (LOH)

Genomic DNA samples extracted from primary glioblastoma oncosphere lines were run on an Affymetrix Genome-wide Human Single Nucleotide Polymorphism (SNP) 6.0 Array in the Johns Hopkins University School of Medicine High Throughput Biology Center Microarray Core facility. The fluorescent intensity values were used by CNViewer and Partek software for copy number variant (CNV) and loss of heterozygosity (LOH) analysis.

### Immunohistochemistry

Formalin-fixed, paraffin-embedded primary glioblastoma tissue samples were analyzed for endogenous L1 ORF1p expression as previously described [28, 29]. Briefly, 5-μm thick sections were deparaffinized and hydrated by baking at 65 °C for 20 min and then with xylene and ethanol washes. Sections were heated at 98 °C in citrate buffer for 20 min for antigen retrieval. Sections were blocked at room temperature for 10 min and then incubated with primary monoclonal mouse L1-ORF1p antibody (1:1000 dilution) in Tris-buffered saline (TBS), pH 7.2 with Tween 20 and 1%BSA overnight at 4 °C. Sections were washed with TBS and incubated with biotin-conjugated anti-mouse IgG for 10 min at room temperature. Sections were developed with 3,3′- Diaminobenzidine (DAB) chromagen mix, counter stained with hematoxylin, dehydrated and coverslipped.

## Results

### Transposon insertion profiling

We obtained seven primary GBM samples from patients 50–65 years old (average age 57) as well as peripheral blood draws from the same individuals. We established oncosphere cultures to expand the tumor cells and extracted high molecular weight genomic DNA from both the oncospheres and peripheral blood mononuclear cells. Primary tissue directly from these resections was available in sufficient quantity to assay directly for four cases. We digested these samples with restriction enzymes, ligated vectorette oligonucleotides to their ends, and selectively amplified LINE-1 genomic insertion sites using vectorette PCR as previously described.

Vectorette PCR is a ligation mediated PCR that allows for the amplification of unknown sequence downstream of a sequence of interest. In this case, the PCR recovers L1Hs or L1(Ta) insertions and the genomic sequence immediately downstream. L1Hs are known to be the most active LINE-1 in modern humans [4]; they are responsible for the most variation in human populations as well as the largest proportion of *de novo* and somatic LINE-1 insertions [3]. The specificity of this PCR for L1Hs is imposed by the position of one of the amplification primers located in the 3′UTR of the LINE-1 elements. The primer positioning is also advantageous because it allows for the recovery of insertions that are severely 5′ truncated, a common feature of LINE-1 insertions.

**Fig. 1:**
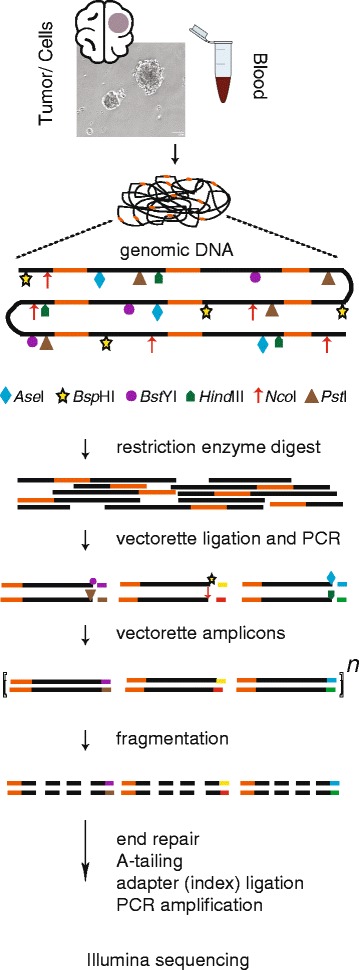
Transposon Insertion Profiling by sequencing (TIPseq) workflow. High molecular weight genomic DNA was extracted from primary and secondary glioblastoma (GBM) tumors, oncosphere cultures expanded from primary GBM, and matched blood samples from the same patients. Genomic DNA was then digested in six parallel reactions each using one of a panel of restriction enzymes. In the diagram, LINE-1 insertions are depicted as orange segments of the genomic DNA; restriction enzyme cuts sites are illustrated with different symbols. Vectorette oligonucleotides designed to match each restriction enzyme sticky end were ligated to the DNA fragments, and the 3′ ends of LINE-1 sequences and downstream DNA were amplified. Genomic DNA fragments without binding sites for the LINE-1 amplification primer are not enriched in this PCR. Amplified DNA was then randomly sheared and prepared for Illumina sequencing

In addition to the primary GBM oncosphere cultures, we also acquired three secondary GBM samples and matching blood samples from patients 33–53 years old (average age 44). These tumors are less readily expanded in vitro, so tissue from the resected tumors was used directly to make genomic DNA for TIPseq in each case. Figure 1 illustrates the TIPseq workflow and Additional file 1: Table S1 summarizes the sequencing batches, TIPseq library indices, and total reads obtained from each sample for this study.

### Germline LINE-1 insertions not found in oncosphere cultures

**Fig. 2:**
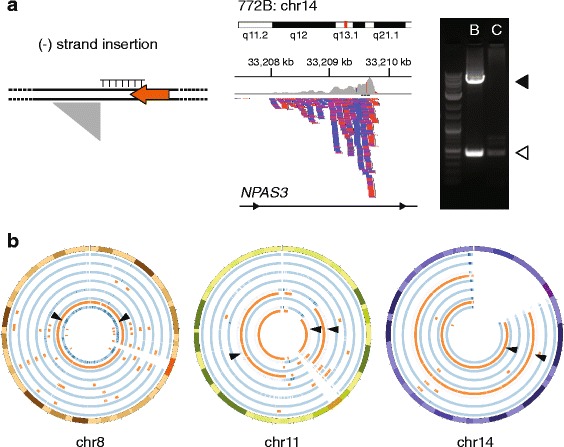
TIPseq in primary glioblastoma GBM oncosphere lines and corresponding blood samples. **a**. TIPseq data. (Leftmost panel) The schematic depicts a minus (-) strand L1 as a leftward facing orange arrow. The LINE-1 sequence ends with a 3′ polyA tail, shown as a homopolymer of thymine (T) on the complementary strand. The gray right triangle illustrates the shape of sequencing reads piling up (*vertically*, *downward*) when mapped against in the reference genome (i.e., with genome coordinates depicted on the horizontal axis). (*Central panel*) TIPseq read alignments corresponding to an insertion detected in blood and not the patient’s oncosphere cell line. The insertion is in an intron of the *NPAS3* gene (14q13.1). Read depth is illustrated on the top (*gray*) and individual reads are represented as blue and red bars denoting orientation. (*Rightmost panel*) An agarose gel electrophoresis of a validation PCR. The open arrowhead (*lower*) marks the pre-insertion allele and the solid arrowhead (upper) marks the amplicon spanning the LINE-1 insertion. The insertion is detected in the blood (B) sample for this patient and not the corresponding tumor cells (C). The LINE-1 is 5.4 kb. **b**. Copy number and loss of heterozygosity (LOH) studies on the oncosphere cell lines. Results for chromosomes 8, 11 and 14 are shown in Circos plots. The seven samples are each depicted as two circular tracks of data. The blue track indicates copy number; medium blue is diploid, darker blue shows amplifications, and lighter blue shows deletions. The orange track highlights regions with LOH. (Leftmost circle) Two insertions on chromosome 8 are marked with arrowheads at 25 and 120 MB; both were identified in a blood sample and not the corresponding patient’s oncosphere cell line, which showed a copy neutral LOH of the entire chromosome (847). (*Central circle*) Three LINE-1 insertions on chromosome 11 found in blood only are marked; two are the same insertion at 33.6 MB found in two different patient samples (847, 922). Both tumor cell lines had deletions with copy number decreases and LOH at this site. One of these cases (847) also had loss of material near 94.3 MB associated with deletion of a second LINE-1. (*Rightmost circle*) Two LINE-1 are marked with arrowheads at 30.2 and 33.2 MB on acrocentric chromosome 14. These were found in genomic DNA from blood, but were lost owing to LOH in the corresponding oncosphere lines (897, 772)

We sequenced 14 TIPseq amplicons preparations from primary GBM oncosphere cultures and matching peripheral blood samples from 7 patients in 3 different sequencing batches. We compared TIPseq profiles from blood and oncosphere samples for each individual, and identified numerous putative LINE-1 insertions in the blood samples that were absent from the corresponding oncosphere cultures. We validated 11 of these by PCR; in 8 we were also able to Sanger sequence the LINE-1 insertion and report both the 5′ and 3′ ends and the target site duplication (Tables 1 and 2, Fig. 2a). Instances of this occurred in six of our seven cases. The size of these LINE-1 elements ranged from full-length insertions of 6059 bp to 5′ truncated insertions as short as 684 bp. Several of these were in gene introns, namely guanylate cyclase 1, soluble, beta 2 pseudogene (*GUCY1B2*), neuronal PAS domain 3 (*NPAS3*), the uncharacterized KIAA159-like gene (KIAA1549L), sterile alpha motif domain 12 antisense RNA-1 (*SAMD12-AS1*), and sec1 family domain 1 (*SCFD1*). None were in exons.

**Table 1:** Candidate L1 insertions tested

Sample ID	Tumor	Blood
Primary glioblastoma		
714	0/19	0/6
750	0/18	**2**/9
772	0/24	**1**/11
832	0/9	**1**/19
847	0/8	**2**/6
897	0/2	**4**/23
922	0/11	**1**/7
Secondary glioblastoma		
007	0/93 + 0/13	0/0
023	0/25	0/0
083	**1**/44	0/0

Number of candidate L1 insertions detected in either tumor or peripheral blood DNA and tested by PCR from putative insertions in primary glioblastoma cell cultures, secondary glioblastoma tissue and matched blood samples

Numerators indicate numbers of productive PCR reactions. Denominators indicate candidate loci tested. Non-zero numerators are in bold

**Table 2:** PCR validated L1 insertions

Sample ID	Chromosome	5′ junction coordinate	3′ junction coordinate	L1 strand	Length of inserted L1	Length of TSD	Gene name	CN	LOH
Blood specific insertions									
750	5	.	34495746	+	.	.	.	1	Y
750	13	50488000	50487984	+	4162 bp	17 bp	*GUCY1B2*	1	Y
772	14	33209876	33209888	-	5422 bp	13 bp	*NPAS3*	1	Y
832	10	31557469	31557476	-	6059 bp	8 bp	.	1	Y
847	11	33626714	33626699	+	1281 bp	16 bp	KIAA1549L	1	Y
847	11	.	94317493	-	.	.	.	1	Y
897	8	24982467	24982461	-	2845 bp	7 bp	.	2	Y
897	8	119790870	119790884	-	965 bp	15 bp	*SAMD12AS1*	2	Y
897	13	85238154	85238150	-	684 bp	5 bp	.	1	Y
897	14	.	30220577	-	.	.	*SCFD1*	2	Y
922	11	33626714	33626699	+	1281 bp	16 bp	KIAA1549L	1	Y
Tumor specific insertions									
083	17	47881841	47881832	+	1839 bp	10 bp	.	NT	NT

List of L1 insertions validated by PCR in glioblastoma patients’ samples

*TSD* target site duplication, *CN* copy number, *LOH* loss of heterozygosity, *NT* not tested

One of these insertions, the LINE-1 at the KIAA1549L locus, was found in two unrelated patient blood samples, although it was absent from the corresponding oncosphere cell lines. Several others, including the insertions at *GUCY1B2*, *NPAS3*, *SAMD12-AS1* and *SCFD1* loci had been previously reported to be polymorphic LINE-1 insertions [30]. Knowing that these LINE-1 insertions are segregating in human populations, we could not attribute the discordance between blood and oncosphere cell culture genotypes to somatically acquired insertions resulting in mosaicism in these patients. Rather, it seemed likely that these represented polymorphic and heterozygous germline copies that were lost in the glioblastoma, e.g. by loss of heterozygosity.

To distinguish between sample contamination and the possibility that these germline insertions had been lost in the GBM cell lines, we assessed copy number and heterozygosity in the oncosphere cell lines using Affymetrix SNP 6.0 microarrays. All LINE-1 insertions that we detected uniquely in blood were indeed located in regions where the corresponding oncosphere cell lines showed loss of heterozygosity (LOH). LOH was seen at some loci with maintenance of copy number (2n) and was seen at others where deletions resulted in reduced copy number (1n) (Table 2, Fig. 2b). We conclude that our findings reflect deletions of genomic LINE-1 associated with LOH events that had occurred either in the primary GBM or *in vitro* as oncosphere cultures of these tumors were established.

### No tumor-specific LINE-1 insertions in primary GBM

We found no evidence of somatically acquired, tumor-specific insertions in primary GBM samples. We compared the seven TIPseq profiles from oncosphere cultures to those from matched peripheral blood gDNA. Additionally, for four of these samples, we had sufficient primary resected tumor to conduct tumor:normal comparisons without *in vitro* GBM expansion. We used an analytical pipeline that ranks peaks by numbers of contributing reads and manually reviewed hundreds of candidates. Although most lacked evidence of junction reads (*i.e.*, reads spanning the 3′ end of the LINE-1 and adjacent, unique genomic sequence), we tested a total of 91 putative tumor-specific insertions in spanning PCR assays and could not detect any with a LINE-1 insertion (Table 1). This is the same approach we used to identify somatically acquired insertions in pancreatic ductal adenocarcinomas and malignancies of the nearby tubular gastrointestinal tract [13].

### Infrequent tumor-specific LINE-1 insertions in secondary GBM

**Fig. 3:**
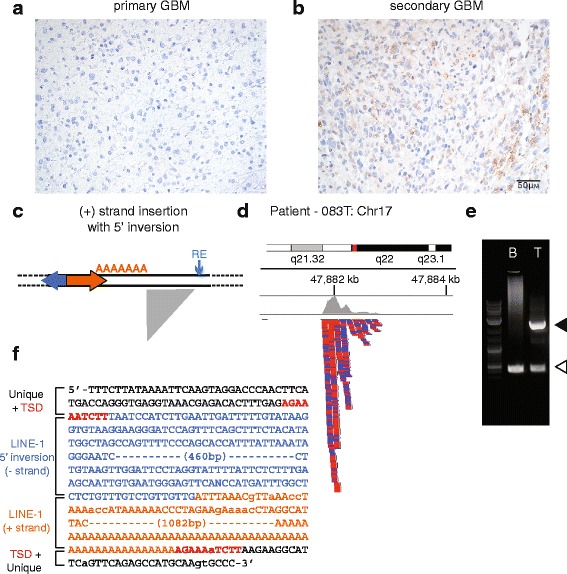
Expression and somatic retrotransposition of LINE-1 in secondary GBM. **a**. and **b**. LINE-1 ORF1p immunohistochemistry. **a**. Most primary GBMs and low grade gliomas do not have detectable LINE-1 ORF1p in this assay. Nuclei are counterstained in blue. **b**. About 74 % of secondary GBM cases are weakly, focally immunoreactive for LINE-1 ORF1p. (*Brown*). **c-f**. Identification of a somatically acquired LINE-1 insertion. **c**. The schematic depicts a plus (+) strand L1 as a rightward facing orange arrow with its 5′ inversion as a leftward facing blue arrow. The genomic LINE-1 sequence ends with a 3′ polyA tail. The gray right triangle illustrates the sequencing reads piling up (*vertically, downward*) when mapped against in the reference genome on the horizontal axis. **d**. TIPseq read alignments corresponding to an insertion detected in a secondary GBM tumor sample and not the patient’s blood DNA. The insertion is an intergenic LINE-1 on chromosome 17q22. Read depth is illustrated on the top (*gray*) and individual reads are stacked downward as blue and red bars denoting orientation. The greatest depth is immediately adjacent to the LINE-1 and extends 3′ of the element to create the triangular shape. **e**. An agarose gel electrophoresis of a validation PCR. The open arrowhead (*lower*) marks the pre-insertion allele and the solid arrowhead (*upper*) marks the amplicon that spans the LINE-1 insertion. The insertion is detected in the tumor (T) sample for this patient and not the corresponding blood cells (B). The LINE-1 is 5′ truncated at 1.8 kb. **f**. The annotated Sanger sequence for the LINE-1 insertion is shown in colored text: flanking unique genomic DNA (*black*), target site duplication (*red*), LINE-1 5′ inversion (*blue*), and LINE-1 3′ sequence and polyA tail (*orange*). Lowercase letters denote lower quality basecalls. These were confirmed by manually examining the trace file

LINE-1 sequences code for two proteins essential for their retrotransposition; these are termed open reading frame 1 and open reading frame 2 proteins (ORF1p and ORF2p). We previously reported that about 33 % of cases in a tissue microarray collection of glioblastomas (GBM) express LINE-1 ORF1p [28]. This was higher than for low grade gliomas. When we distinguished between primary and secondary GBM cases in this study, it was clear that this frequency reflected the numbers of secondary GBM cases included in our survey. These secondary GBMs showed the greatest proportion of LINE-1 ORF1p positivity by immunohistochemistry (74 %, *n* = 39). Although many cases showed weak immunoreactivity, we viewed this as evidence that perhaps these secondary GBM tumors would be relatively permissive for retrotransposition (Fig. 3a-b).

To test secondary GBM for somatically acquired LINE-1 integrations, we obtained 3 tumor samples from neurosurgical resections and matched peripheral blood DNA from the same individuals. We analyzed these sequencing data using two approaches. For the first, we used the same strategy described above which ranks peaks on the basis of the numbers of contributing reads. We manually reviewed these and identified a total of 162 putative tumor specific somatic insertions carried forward to validation studies (Table 1). Although most lacked evidence of junction reads (*i.e.*, reads spanning the 3′ end of the LINE-1 and adjacent, unique genomic sequence), we tested these in spanning PCR assays.

We validated a single tumor-specific insertion at 17q22. Sanger sequencing showed that this insertion had all of the features of a LINE-1 retrotransposed by target primed reverse transcription (TPRT). The element has an intact 3′ polyA tail and 3′ LINE-1 sequence and is flanked by a 10 bp target site duplication. The insertion is 5′ truncated to a length of 1839 bp, which includes a 662 bp inversion of its 5′ end (Fig. 3c-f). These features are characteristic of many somatically acquired LINE-1 insertions [13]. The insertion is intergenic, and to our knowledge, no heritable or somatically acquired element variants have been reported at this position.

To confirm this finding and more thoroughly review the entirety of these data, we also analyzed these sequences using a more advanced machine learning based approach. This algorithm combines five types of information at each locus to identify insertions. The pipeline imposes requirements for 3′ LINE-1 sequence and incorporates metrics to reflect the quantity, quality, and distribution of read alignments to the reference genome as well as measures of polyA tail purity and the numbers of junction reads. It is trained on known LINE-1 insertions recovered within the same run of the same sample, and then used to predict other insertions. The outcome was 26 low probability insertions called in one sample (007); no predicted somatic insertions in the second sample (029); and only the chr17q22 insertion in the third sample (083). For samples 029 and 083, this outcome agreed perfectly with our previous PCR validations. In light of these new predictions for sample 007, we designed 13 additional pairs of spanning PCRs to test half of the 26 putative insertions. Gel electrophoresis of these PCR products provided no support for somatically acquired transposition events. All amplicons matched the predicted sizes for an empty site.

Thus, although we examined only a few cases of secondary GBM, our data suggest that somatic LINE-1 retrotransposition is not prominent in these malignancies.

## Discussion

Somatic retrotransposition of LINE-1 leading to cancer was first described by Miki *et al.* in 1992 [31]. It would take more than 20 years to demonstrate the potential of next generation sequencing technologies to find such events [6]. Since that time, progress has been quick, with both targeted and whole genome sequencing demonstrating LINE-1 instability in a large number of human cancers. Chief among these has been the gastrointestinal tract tumors, including colon [10, 12, 15], esophagus [32, 33], hepatocellular carcinomas [11], and pancreatic ductal adenocarcinomas [13]. Lung and ovarian cancers also demonstrate LINE-1 retrotransposition [6, 14, 17, 18]. In contrast, surveys of selected hematolymphoid tumors and glioblastomas have indicated that these malignancies are not as prone to somatic LINE-1 reintegration [14].

To test this conclusion using a targeted sequencing approach, we profiled LINE-1 insertion sites in 10 cases of GBM. Our samples included 7 primary GBM cases; each was represented by an oncosphere cell line, and 4 primary GBM tissue biopsies were also assayed. We also profiled LINE-1 insertions in 3 secondary GBM tissue samples. In each case, normal brain tissue was not available for comparison, and peripheral blood draws were used to infer the normal genetic make-up of LINE-1 in each of these individuals.

We found no evidence of somatically acquired LINE-1 insertions in primary GBM cases in this study. We do not think that this is attributable to limitations of our assay. In previous studies, the same assay and analyses in our hands have been effective in detecting somatic LINE-1 insertion [13] (Zuojian Tang, et al. in review). Indeed, in this study, our approach was effective in identifying LINE-1 insertions deleted in loss-of-heterozygosity events, which effectively shows that we can detect elements present in one sample and absent from the other. Similar targeted sequencing studies, performed by an orthogonal method, reported in *Mobile DNA* by the Faulkner laboratory also reveal no canonical LINE-1 retrotransposition events (Carreira, et al. MDNA-D-16-00017).

Our work does suggest an interesting distinction between primary and secondary GBM. Unlike primary GBM, a majority of secondary GBM cases show some immunoreactivity for the LINE-1 encoded RNA binding protein ORF1p. Here, we also report finding a single, apparently somatically-acquired LINE-1 insertion in a case of secondary GBM. This insertion has several sequence features to indicate it resulted from a canonical, LINE-1 retrotransposition event. It is a 5′ truncated LINE-1 insertion, with a 5′ inversion and a 3′ polyA tail; the insertion is flanked by a short target site duplication.

When this LINE-1 was acquired is an open question. Our finding of increased ORF1p in secondary GBM implies that these tumors may provide a cellular context permissive for LINE-1 expression and retrotransposition that is unlike the normal adult brain or primary high grade gliomas. Although we favor this possibility, there is also evidence for somatic retrotransposition in the central nervous system as well as genetic variation within the brain reflecting retrotransposition events in early development. Since in all cases, we used blood as the germline comparison, genetic mosaicism antedating tumor initiation cannot be excluded. In either scenario, we presume that this LINE-1 integration has had no direct role in promoting tumor development in this case; it is an intergenic insertion several tens of kilobases away from the nearest gene and in a location with no recognized significance for the development of brain cancer.

## Conclusions

Our findings indicate that LINE-1 retrotransposon events are infrequent in glioblastomas. While examples of driving mutations mediated by target-primed reverse transcription (TPRT) are being recognized in some types of malignancies, we expect these to be relatively uncommon in glioblastoma.

## Additional file

Additional file 1: Table S1. Sequencing information and statistics. (XLS 33 kb)

## Abbreviations

CNV: Copy number variant
GBM: Glioblastoma
LINE-1 or L1: Long INterspersed element-1
LOH: Loss of heterozygosity
ORF1p: Open reading frame 1 protein
SNP: Single nucleotide polymorphism
TIPseq: Transposon insertion profiling by sequencing
TPRT: Target primed reverse transcription
UTR: Untranslated region
